# Highly Pathogenic Avian Influenza Virus A(H5N1) Clade 2.3.4.4b Infection in Free-Ranging Polar Bear, Alaska, USA

**DOI:** 10.3201/eid3008.240481

**Published:** 2024-08

**Authors:** Raphaela Stimmelmayr, David Rotstein, Mia Kim Torchetti, Robert Gerlach

**Affiliations:** North Slope Borough, Utqiagvik, Alaska, USA (R. Stimmelmayr);; University of Alaska, Fairbanks, Alaska, USA (R. Stimmelmayr);; Marine Mammal Pathology Services, Olney, Maryland, USA (D. Rotstein);; US Department of Agriculture Animal and Plant Health Inspection Service, Ames, Iowa, USA (M.K. Torchetti);; Alaska Department of Environmental Conservation, Anchorage, Alaska, USA (R. Gerlach)

**Keywords:** highly pathogenic avian influenza virus, HPAI, clade 2.3.4.4b, influenza, polar bear, subsistence, respiratory infections, viruses, zoonoses, Alaska, United States

## Abstract

We report a natural infection with a Eurasian highly pathogenic avian influenza A(H5N1) clade 2.3.4.4b virus in a free-ranging juvenile polar bear (*Ursus maritimus*) found dead in North Slope Borough, Alaska, USA. Continued community and hunter-based participation in wildlife health surveillance is key to detecting emerging pathogens in the Arctic.

Since its emergence in Europe during October 2020, highly pathogenic avian influenza (HPAI) A(H5N1) clade 2.3.4.4b virus has frequently spilled over into diverse mammal hosts globally. In North America, natural H5N1 infections have occurred in several bear species, including American black bears (*Ursus americanus*), Asiatic black bears (*U. thibetanus)*, grizzly bears (*U. arctos horribilis*), and Kodiak brown bears (*U. a. middendorffi*) (*1*). Infections with influenza A(H1N1) viruses have been reported in captive sloth bears (*Melursus ursinus*) and Asiatic black bears (*2*,*3*) and in giant pandas (*Ailuropoda melanoleuca*) (*4*). Detection of hemagglutination inhibition antibodies against H3 and H6 subtype influenza viruses also suggested previous natural exposure to influenza viruses of avian origin (*4*). Seroconversion after natural exposure to bird influenza viruses has been documented in the Barent Sea polar bear subpopulation (2010–2011) (*5*) and brown bears in Alaska (2013–2016) (*6*) but not in the southern Beaufort Sea polar bear subpopulation (2013–2016) (*7*). Polar bears are a threatened species under the US Endangered Species Act. We report and describe an infection by HPAI H5N1 virus in a free-ranging polar bear found dead in Alaska, USA, during 2023. 

## The Study

The North Slope Borough Department of Wildlife Management (NSB DWM) in Alaska conducts wildlife health research and maintains community-based harvest monitoring programs for marine mammals, including polar bears. The Alaska Office of the State Veterinarian conducts surveillance for notifiable infectious diseases in wildlife. After detecting HPAI H5N1 in birds of prey and a red fox (*Vulpes vulpes*) in April 2022, the Office of the State Veterinarian initiated collaborative surveillance testing with NSB DWM for avian influenza in birds and other wildlife. In August 2023, community members reported a dead polar bear without obvious external injuries near Point Barrow, Alaska (71°23′N, 156°28′W). At the NSB DWM laboratory in Utqiagvik, Alaska, we conducted a postmortem examination of the bear. The bear was young and male, 120 cm in body length, and in moderate to advanced decomposition. Body condition was fair to poor, with no back or visceral fat. Gross findings were multiple 1–3-cm ulcerative skin lesions around the left eye and oral commissure, liver and lung congestion, moderate sanguinal pericardial and cavitary effusion, cerebral swelling and congestion, and empty stomach. We collected postmortem tissue samples of the heart, lung, trachea, spleen, liver, kidney, adrenal gland, skin, skeletal muscle, mesenteric lymph node, pancreas, tongue, esophagus, stomach, small intestines, and brain (cerebrum) and fixed them in 10% neutral buffered formalin for 2 weeks. Histology Consultation Services (https://histocs.com) processed the tissue for routine histopathologic examination by staining with hematoxylin and eosin. We also collected oral, nasal, rectal, and brain swab samples and placed them in 2-mL cryovials, which we stored for 2 weeks at −50°C and shipped to the Alaska Environmental Health Laboratory in Anchorage, Alaska. Their personnel placed pooled swab specimens into brain–heart infusion broth. The primary histopathologic finding was a granulocytic and mononuclear meningoencephalitis with microgliosis, neuronal necrosis, neuronophagia, vasculitis, and parenchymal rarefaction (Figure, panel A). Other findings were pulmonary edema, focal lipid pneumonia, and multifocal ulcerative dermatitis. 

**Figure:**
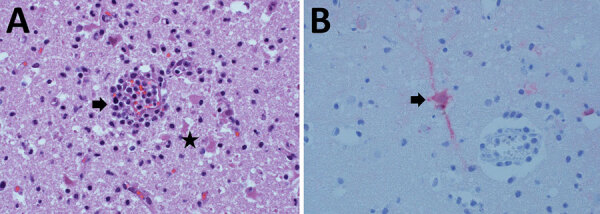
Histologic analysis of brain tissue from a dead free-ranging polar bear infected with highly pathogenic avian influenza virus A(H5N1) clade 2.3.4.4b, Alaska, USA. A) Hematoxylin and eosin staining of brain tissue section showing meningoencephalitis. Arrow indicates mixed inflammatory cells within and around blood vessels and hypertrophied vascular endothelial cells. Star indicates necrotic neurons and increased number of microglial cells within the parenchyma. Original magnification ×400. B) Arrow indicates influenza A virus within the neuronal perikaryon (red staining) observed by immunohistochemistry of formalin-fixed paraffin-embedded brain sections. Original magnification ×400.

Pooled swab specimens tested negative for the influenza virus matrix gene by PCR at the Washington Animal Disease Diagnostic Laboratory (Pullmans, WA, USA), which is a National Animal Health Laboratory Network facility. The root cause of negative PCR results is unclear because subsequent sequence analysis did not indicate assay failure. However, because of the cerebral lesions, we sent scrolls of formalin-fixed paraffin-embedded cerebral tissue to the Athens Disease Diagnostic Laboratory, University of Georgia (Athens, GA, USA), for immunohistochemistry to detect influenza A by using an influenza A virus polyclonal antibody (Abcam, https://www.abcam.com). Influenza A virus antigen was detected in cytoplasm of neurons and nuclei of microglial cells (Figure, panel B). We also sent scrolls of formalin-fixed paraffin-embedded cerebral tissue to the National Veterinary Services Laboratories (Ames, IA, USA), for molecular confirmation and virus genome characterization. HPAI virus genotype A3, a fully Eurasian influenza virus, was identified; this genotype was initially detected in Alaska in April 2022 and was the most frequently detected genotype in Alaska during August–December 2023. Reported markers for mammal adaptation were not identified. We deposited full genome sequences for the polar bear virus (A/polar bear/Alaska/23–0381234/2023) in GenBank (accession nos. PP820319–26) and GISAID (https://www.gisaid.org; accession no. EPI_ISL_18976667).

This detection of HPAI virus was in the southern Beaufort Sea subpopulation, 1 of 19 circumpolar polar bear subpopulations. During July–August 2023, three short-tailed shearwater seabirds (*Ardenna tenuirostris*) that tested positive for HPAI H5N1 clade 2.3.4.4b were found dead near Point Barrow, where the polar bear in this study was found. The shearwaters’ virus genotype shared 9 common single-nucleotide polymorphisms (SNPs) with the polar bear virus and was representative of the virus circulating in that area at the time rather than a direct source of the polar bear infection. In addition, in August 2023, a small mortality event from avian influenza occurred among common murre seabirds (*Uria aalge*) in Dillingham Census Area in Alaska; that virus genotype also shared 8–9 common SNPs, further supporting regional virus circulation. Polar bears are primarily dependent on seals as a food source but will prey on birds and eggs; thus, virus exposure from consumption of infected birds is possible, but infection via an olfactory route cannot be excluded (*8*). 

Support does not exist for ongoing HPAI virus–associated illness and death in free-ranging polar bears in Alaska’s North Slope Borough; in 2023, swab specimens from 3 other dead polar bears tested negative for influenza virus by PCR (Appendix). As for black bears with HPAI H5N1 virus infections (*9*), brain lesions were the major histopathologic findings in this case. It is not unexpected for clade 2.3.4.4b virus–infected mammals with neurologic signs to have respiratory samples test negative (*10*), possibly because of different exposure routes, such as digestive, olfactory, or respiratory routes (*8*). The HPAI H5 goose/Guangdong lineage has been shown to be more neuropathogenic than other influenza A viruses in mammals (*8*), including the H5N1 clade 2.3.4.4b virus (*11*,*12*). 

Other notable findings in this case were pulmonary edema, lipid pneumonia, and ulcerative skin lesions. Pulmonary edema as a gross lesion and on histologic analysis was a consistent finding in 3 domestic cats with H5N1 clade 2.3.4.4b infections (*13*) but has been infrequently reported in wild mesocarnivores (*10*). Lipid pneumonia was documented in subsistence-harvested polar bears in the southern Beaufort Sea and is considered unrelated (D. Rotstein, unpub. data). Ulcerative skin lesions not caused by trauma are rarely documented in subsistence-harvested southern Beaufort Sea polar bears (R. Stimmelmayr, unpub. data); those lesions have not been reported in terrestrial mammals infected with the H5 clade 2.3.4.4b lineage (*10*) but have been reported in pinnipeds infected with H3N8 virus (*14*). 

## Conclusions

Genome analysis of influenza viruses originating from wildlife in Alaska has shown both unreassorted and reassorted viruses. HPAI virus genotype A3 was likely introduced into Alaska via the East Asia–Australia Flyway as early as November 2021 (*15*) and has been detected in a few backyard premises; in many wild birds, including California condors (*Gymnogyps californianus*) in Arizona; and several mammals (red fox, fishers, martens, racoons, and brown bears) along the Pacific Flyway.

In the Arctic, wildlife and other wild subsistence foods play a pivotal role in the health, well-being, and food security of northern indigenous communities. Therefore, subsistence harvesting of animals infected with HPAI viruses, including polar bears, poses a zoonotic risk and affects traditional food safety and food security. Continued community and hunter-based participation in wildlife health surveillance is key to detecting emerging pathogens and other One Health issues in the Arctic.

Appendix Additional information for highly pathogenic avian influenza virus A(H5N1) clade 2.3.4.4b infection in free-ranging polar bear, Alaska, USA.
