# Supplementary material for: Highly Pathogenic Avian Influenza Virus A(H5N1) Clade 2.3.4.4b Infection in Free-Ranging Polar Bear, Alaska, USA
Source: Emerg Infect Dis. 2024 Aug;30(8):1660–3. doi: 10.3201/eid3008.240481 (PMC11286074; doi:10.3201/eid3008.240481)
Supplement: Appendix — Additional information for highly pathogenic avian influenza virus A(H5N1) clade 2.3.4.4b infection in free-ranging polar bear, Alaska, USA. [file 24-0481-Techapp-s1.pdf]

Article DOI: https://doi.org/10.3201/eid3008.240481
EID cannot ensure accessibility for supplementary materials supplied by authors. Readers who have difficulty accessing supplementary content should contact the authors for assistance.

Highly Pathogenic Avian Influenza Virus A(H5N1) Clade 2.3.4.4b Infection in Free-Ranging Polar Bear, Alaska, USA

Appendix

Additional Methods

2023 Polar Bear Necropsies, North Slope Borough Department of Wildlife Management

A summary of avian influenza surveillance findings from 3 other polar bears found dead and 1 defense-of-life kill near Utqiagvik, Alaska, USA, collected during August–December 2023 is provided. Three of the 4 bears (polar bears no. 1–3) were found dead in the surf near Utqiagvik and 1 bear (polar bear no. 4) was a defense-of-life kill in Utqiagvik. No clinical signs of illness in polar bear number 4 were observed. Indigenous hunters salvaged the heads from polar bears 2 and 3. Postmortem examination of polar bear 1 (only external) and polar bear 4 (complete) was conducted by the wildlife veterinarian from the North Slope Borough Department of Wildlife Management. Tissue samples for routine histopathologic examination were fixed in 10% neutral-buffered formalin and processed for hematoxylin and eosin staining at the Histology Consultation Services (https://histocs.com) in Everson, Washington, USA. Swab specimens (i.e., oral, nasal, rectal, brain) were collected by using polyester swabs on plastic stems, placed in 2 mL cryovials, and stored frozen at –50°C. At the Alaska Environmental Health Laboratory, swab specimens were pooled and placed in brain heart infusion broth before sending out for analysis.

PCR

Swab specimens were tested by PCR of the influenza A virus matrix gene at the Washington Animal Disease Diagnostic Laboratory, a member of the National Animal Health Laboratory Network (NAHLN). Members of NAHLN use the same PCR protocol as the National Veterinary Services Laboratories in Ames, Iowa. The subsequent sequence analysis for the highly pathogenic avian influenza virus–positive polar bear in this study did not point to any

issues with the PCR itself; therefore, we cannot resolve the root cause for the initial negative PCR test results for swab specimens. However, it is reasonable to conclude that the negative PCR results from swab specimens from the additional 3 polar bears found dead (see below) reflect true negatives and support ruling out suspicion of HPAI infection in those 3 bears.

IHC

Influenza A virus immunohistochemistry was performed by staining formalin-fixed paraffin-embedded tissue sections with influenza A virus polyclonal antibody (Abcam, https://www.abcam.com). Immunohistochemistry was performed by the University of Georgia, Athens Disease Diagnostic Laboratory (a member of NAHLN).

Polar Bear 1 (ID: 2023PB1007FDA; Collection date: 7-Oct-2023)

An adult, male polar bear carcass in advanced decomposition was found; snout and claws were salvaged. Body condition was excellent. Because of advanced decomposition, only skin and skeletal muscle were collected for histopathologic examination. No evidence of inflammation, necrosis, or infectious agents was observed. Swab specimens (brain [spinal cord], rectal, nasal) tested negative for influenza virus by PCR.

Polar Bear 2 (ID: 2023PB1007FDB; Collection date: 7-Oct-2023)

A subadult, male polar bear carcass in moderate decomposition was found. Body condition was good and no external injuries or gunshots were noted by the hunter. Brain (spinal cord) and skeletal muscle were collected from the salvaged head. Histopathologic examination showed a few myofibers contained nematodes (*Trichinella* spp.). No evidence of inflammation, necrosis, hemorrhage, vasculitis, or infectious agents was observed in the brain. Swab specimens (brain, nasal) tested negative for influenza virus by PCR.

Polar Bear 3 (ID: 2023PBRexford; Collection date: 25-August-2023)

A subadult, male polar bear carcass in advanced decomposition was found. No external injuries or gunshots were noted by the hunter. Only skin and skeletal muscle were collected for histopathologic examination. No evidence of inflammation, necrosis, or infectious agents was observed. Swab specimens (brain, nasal) tested negative for influenza virus by PCR.

Polar Bear 4 (ID: 2023PB1204DL; Collection date: 4-Dec-2023)

Polar bear 4, a subadult, male in excellent body condition, was alive and exhibited aggression toward vehicles. Deterrence efforts by North Slope Borough polar bear patrol staff

were unsuccessful, and the bear was killed by two gunshots to the head. Gross and histopathologic findings showed trauma to the costochondral joints (ribs 2–6) with focal muscle necrosis of the adjacent muscles, lipid pneumonia, hepatic and splenic pallor (hypovolemia), and a focal chronic renal infarct. Brain tissue could not be examined because of the kill method. Bird feathers with other food items (bowhead whale blubber and meat) were observed in the gastrointestinal tract. No infectious agents were observed in the lungs. Because of the bird feathers in the gastrointestinal tract and the aggressive antemortem behavior of the polar bear, influenza A virus immunohistochemistry was performed on formalin-fixed paraffin-embedded sections of lung; influenza A staining was negative.
